# Validating MODIS and Sentinel-2 NDVI Products at a Temperate Deciduous Forest Site Using Two Independent Ground-Based Sensors

**DOI:** 10.3390/s17081855

**Published:** 2017-08-11

**Authors:** Maximilian Lange, Benjamin Dechant, Corinna Rebmann, Michael Vohland, Matthias Cuntz, Daniel Doktor

**Affiliations:** 1 Department Computational Landscape Ecology, Helmholtz-Centre for Environmental Research-UFZ, Permoserstr. 15, 04318 Leipzig, Germany; benjamin.dechant@ufz.de (B.D.); daniel.doktor@ufz.de (D.D.); 2 Department Computational Hydrosystems, Helmholtz-Centre for Environmental Research-UFZ, Permoserstr. 15, 04318 Leipzig, Germany; corinna.rebmann@ufz.de (C.R.); matthias.cuntz@ufz.de (M.C.); 3 Leipzig University, Institute for Geography, Geoinformatics and Remote Sensing, Johannisallee 19a, 04103 Leipzig, Germany; michael.vohland@uni-leipzig.de; 4 INRA, Université de Lorraine, UMR1137 Ecologie et Ecophysiologie Forestière, 54280 Champenoux, France

**Keywords:** sensor, hyperspectral, multispectral, validation, site, automatic, measurements, vegetation, MODIS, Sentinel-2, phenology

## Abstract

Quantifying the accuracy of remote sensing products is a timely endeavor given the rapid increase in Earth observation missions. A validation site for Sentinel-2 products was hence established in central Germany. Automatic multispectral and hyperspectral sensor systems were installed in parallel with an existing eddy covariance flux tower, providing spectral information of the vegetation present at high temporal resolution. Normalized Difference Vegetation Index (NDVI) values from ground-based hyperspectral and multispectral sensors were compared with NDVI products derived from Sentinel-2A and Moderate-resolution Imaging Spectroradiometer (MODIS). The influence of different spatial and temporal resolutions was assessed. High correlations and similar phenological patterns between in situ and satellite-based NDVI time series demonstrated the reliability of satellite-based phenological metrics. Sentinel-2-derived metrics showed better agreement with in situ measurements than MODIS-derived metrics. Dynamic filtering with the best index slope extraction algorithm was nevertheless beneficial for Sentinel-2 NDVI time series despite the availability of quality information from the atmospheric correction procedure.

## 1. Introduction

Quantifying the accuracy of satellite-based remote sensing products is an important task that is increasingly timely given the rising number of Earth observation missions. Different measurement strategies are pursued at established validation sites, including continuous in situ (spectral) measurements, airborne remote sensing campaigns at the landscape level, as well as the collection of independent satellite sensor data [1,2]. Linking small-scale observations with large-scale satellite products is crucial to understand the relationships between optical information and ecological, as well as physiological processes [3]. This thus facilitates prediction and mapping of processes at regional and global scales, such as terrestrial carbon assimilation [4]. Automatic continuous spectral measurements are bridging the gap between continuous micro-meteorological measurements and remote-sensing products by providing optical information about vegetation processes with high temporal resolution [5,6]. They are thus helpful to improve the understanding of satellite measurements.

Phenology plays a central role in the estimation of vegetation-dependent processes [7]. Research on vegetation phenology uses phenological and spectral ground observation networks, phenological modeling, eddy covariance towers and satellite-derived imagery to assess and monitor vegetation status and dynamics [8,9]. Several studies are dealing with the question of which vegetation index to choose for satellite phenology depiction [10]. Although the Normalized Difference Vegetation Index (NDVI) shows saturation effects in dense forest canopies [11], it is one of the most widely-used indices for this purpose, mainly due to data availability and its robustness against noise and varying illumination geometries [10,12]. A large number of studies [13,14,15,16] mapped land surface phenology from satellite NDVI data. These studies outlined two main problems in the derivation of phenology from satellite data: firstly, the low spatial resolution of satellite data, e.g., from the NOAA Advanced Very High Resolution Radiometer (AVHRR) and the NASA Moderate-resolution Imaging Spectroradiometer (MODIS). This results in mixed pixels, integrating phenological signals of different vegetation and land cover types, e.g., deciduous forests and nearby grasslands. New satellite generations, such as the Copernicus Sentinel-2, may overcome this issue with higher spatial resolution. Secondly, non-vegetational effects may alter the received phenological signal. These effects include atmospheric conditions, snow cover [11,17], soil-wetness [8], viewing geometry and illumination conditions [18,19], as well as the distorted signal under overcast conditions. Noise introduced by these effects can be eliminated by using methods like Maximum Value Composite (MVC) [20] or using dynamic filtering, such as the Best Index Slope Extraction (BISE) [21]. While MVC is effective at reducing cloud and viewing condition effects, it can include outliers with higher NDVI. Short-term vegetation-state changes might be masked using a long composition period, while a short period retains noise [21]. BISE excludes outliers, and its parametrization options allow for the removal of sudden decreases in NDVI, mainly due to non-vegetational effects. This, however, also excludes sudden decreases in NDVI due to vegetational processes, followed by rapid regrowth [21]. Several approaches are used to fill gaps and smooth the phenological time series after the removal of noisy measurements: interpolation methods, signal filter or model fitting algorithms [12]. Subsequently, phenological metrics are extracted using different approaches: global or local thresholds, conceptual-mathematical models [8] or local extrema of transition rates [22]. Here, we used BISE to filter NDVI data, followed by a simple linear interpolation to remove gaps in the time series, and finally, we extracted phenological metrics with a local threshold method.

The aim of this study is to validate different satellite phenology products by comparing them to multi-sensor ground measurements. This also requires a detailed description of the site’s instrumentation, as well as information on calibration, but the main focus of this study is not on the latter. Details on calibration will be described in a separate publication [23]. In the following, we briefly introduce measurement approaches commonly applied at spectral ground observation sites.

Compact multispectral sensors such as the SKYE SKR1860 series (Skye Instruments Ltd., Llandrindod Wells, Powys, U.K.) are comparably low-cost and fairly straightforward to set up and maintain [6]. Typically, no optical fibers are required, and data can be logged with data loggers commonly used at eddy covariance sites. Therefore, this type of instrument is widely used [24]. Hyperspectral systems, in contrast, are usually more expensive and require computers to control the timing of measurements and instrument settings [6]. Furthermore, it is common that fiber optics are used to connect the spectrometer to the point of measurement [25,26,27], and this introduces additional potential for calibration issues on top of spectrometer calibration itself [6]. However, they provide more detailed spectral information. Both sensor types are potentially affected by issues of long-term continuous field measurements [5,6], such as degradation and sensitivity to temperature and humidity. While other studies focused mainly on either hyperspectral [24,25,26,28] or multispectral sensors [4,19], we used both types of sensors in order to assess measurement quality issues introduced by environmental conditions and instrument settings [24], which might be relevant for the validation of satellite-derived phenological profiles.

In order to compute a reflectance factor, later used for the calculation of NDVI, it is necessary to measure the upwelling and downwelling radiation fluxes. This can be achieved with different setups [6]: systems using one sensor have to measure a reference in sequence to each measurement of the target surface, e.g., a white reference panel [27] or downwelling irradiance through the rotation of the fore optics of the sensor [28]. This approach introduces considerable time delays between downwelling irradiance and upwelling radiance measurements and therefore increases measurement uncertainties, particularly under unstable illumination conditions [27]. The moving parts are an additional potential error source in a long-term outdoor setup. Furthermore, the approach is restricted to a single fore optic for both target and reference measurements, which is suitable only for measurements of bi-hemispherical reflectance factors in practice.

Measuring downwelling irradiance and upwelling radiance quasi-simultaneously with a dual-field-of-view (DFOV) setup and different FOV fore optics [19,24,25] removes the restriction of observing bi-hemispherical reflectance factors. Furthermore, simultaneous acquisition for both FOVs is possible in the case of the use of two sensors, while the time delay depends on sensor characteristics and instrument settings such as integration time if only one sensor is used [26]. However, challenges due to spectral shifts caused by bifurcated fibers (one sensor) and cross-calibration of wavelength scale (two sensors) may arise for DFOV hyperspectral measurements. We used the DFOV approach with two sensors for multispectral measurements and with a single sensor and a bifurcated fiber for hyperspectral measurements. Hyperspectral- and multispectral-based NDVI values (spatial extent on the order of 10 m) were compared with NDVI products derived from MODIS Aqua and Terra (250-m ground resolution), as well as Sentinel-2 (10- and 20-m ground resolution) over the course of two vegetation periods. We evaluated whether dynamic filtering procedures, commonly used with NOAA AVHRR and MODIS, are now obsolete with new generation satellite data. Finally, the influence of varying spatial resolutions of the different datasets on extracted phenological metrics was also assessed.

## 2. Materials and Methods

### 2.1. Study Site and Sensor Setup

We collected data at the forest site ‘Hohes Holz’ (52.08${}^{\circ }$ N 11.22${}^{\circ }$ E), which is situated in central Germany near the Harz Mountains in a temperate climate [29]. The site is part of the interdisciplinary and long-term research programs Tereno (Terrestrial Environmental Observatories [30]) and ICOS (Integrated Carbon Observation System [31]). The ecosystem of ‘Hohes Holz’ is a deciduous forest with a size of around 15 km${}^{2}$. The vegetation within one MODIS pixel around the tower consisted of 50% oak, 45% beech, 2.5% birch and 2.5% clearings, whereas the area within 30 m around the tower had a higher amount of clearings (10%) and birch (4%) and less oak (44%) and beech (42%). The eddy flux tower has a height of 50 m, which is about 20 m above the canopy. We installed one hyperspectral sensor and two multispectral sensors at the site: a QE65000 (Ocean Optics, Dunedin, FL, USA) and an SKR1850 4-channel sensors (Skye Instruments Ltd., Llandrindod Wells, Powys, U.K.).

Data logging of the multispectral sensors is performed with a Campbell Scientific CR1000 data logger with a sampling frequency of 0.1 Hz. The upward facing multispectral sensor is set up with a hemispherical cosine diffuser. The downward facing sensor is used for narrow-angle measurement (25${}^{\circ }$ FOV) of vegetation with a south-orientated (view azimuth of 180${}^{\circ }$) off-nadir view angle of 22.5${}^{\circ }$. Central wavelengths and the full-width at half-maximum (FWHM) were chosen according to Sentinel-2 Channels 4–6 and 8a (see Table 1).

**Figure 1:**
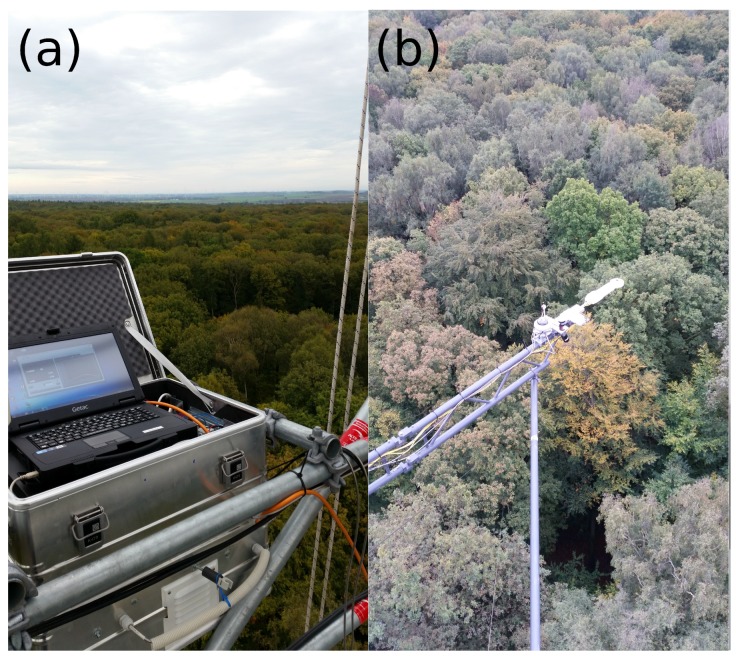
The weather-proof box is mounted at the outside of the eddy flux tower (**a**). The multispectral sensor and optics of the hyperspectral sensor are mounted on a boom of the tower at 49-m height (**b**).

The hyperspectral sensor uses scientific-grade back-thinned detectors (S7031-1006, Hamamatsu Photonics, Hamamatsu City, Shizuoka Pref., Japan) and was set up with a spectrometer slit width of 10 μm, allowing an FWHM of 1.58 nm and a spectrometer grating allowing for observations in the range of 398 nm–1174 nm with 1030 channels (QE65000, Ocean Optics, Dunedin, FL, USA). A 400-μm bifurcated fiber optic cable (QBIF400-VI, Ocean Optics, Dunedin, FL, USA) is used as the dual input channel, where each of the two cable ends is connected with a shutter (INLINE-TTL, Ocean Optics, Dunedin, FL, USA). Each shutter is connected with a 400-μm fiber optic cable (QP400-15-V, Ocean Optics, Dunedin, FL, USA). A cosine corrector (CC-3-UV-S, Ocean Optics, Dunedin, FL, USA) is attached to the upward facing cable end for irradiance measurements. The downward facing cable end is used without fore optics with a field of view of 25${}^{\circ }$ to measure upwelling radiances. The optics are mounted 20 m above the canopy with a south-orientated (view azimuth of 180${}^{\circ }$) off-nadir view angle of 22.5${}^{\circ }$ (see Figure 1b) on a boom of the eddy flux tower. This results in an observed canopy area of around 72 m${}^{2}$.

**Figure 2:**
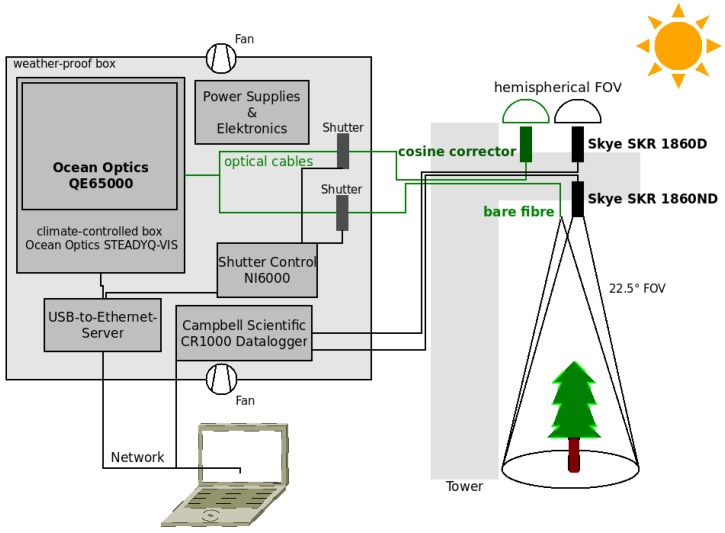
Schematic overview of sensor setup for unattended hyperspectral and multispectral measurements. Electronics and the hyperspectral sensor are installed in a weather-proof box and connected to the network. Fore optics and multispectral sensors are mounted on a boom of a tower above the forest.

The sensor and shutters are controlled via LabView (National Instruments, Austin, TX, USA). Shutters open and close alternately to select the signal from upward- and downward-facing fibers to measure downwelling irradiance and upwelling radiance sequentially. Integration time was set to two seconds between April and September and four seconds between October and March. Dark current is determined with closed shutters before and after each measurement. This procedure is repeated three times every ten minutes and takes 24 seconds (three times eight seconds) between April and September and 48 seconds (three times 16 seconds) between October and March. The whole setup (see Figure 2) is situated in a weather-proof box (see Figure 1a) with a temperature-driven ventilation system. USB devices are connected to a USB-to-Ethernet server to enable remote control. The data logger is connected with Ethernet, and data are transferred automatically once a day onto a network storage system.

### 2.2. Generation of NDVI Products from Ground-Based Spectral Measurements

Hyperspectral solar irradiance data were cross-calibrated once by using measurements of solar irradiance with an ASD FieldSpec 4 spectrometer (Analytical Spectral Devices Inc., Boulder, CO, USA) and a Spectralon panel. Calibration performance was assessed by resampling the QE65000 spectrum to the lower spectral resolution of ASD FieldSpec. High accuracy and precision were achieved when comparing the corresponding two spectra of solar irradiance ($r^{2}=0.99$, relative RMSE=0.03 in the spectral range 440–1000 nm). Hyperspectral upwelling radiance observations were calibrated by using the downwelling irradiance calibration and a Spectralon panel. The stability of wavelength positions was irregularly checked with a mercury argon calibration light source (HG-1, Ocean Optics, Dunedin, FL, USA). Multispectral data were radiometrically corrected by using the channel sensitivities given by the manufacturer. Manufacturer calibration was repeated every two years (2014 and 2016). Long-term stability was assessed by comparing against shortwave pyranometer measurements (CNR4 net radiometer, Kipp&Zonen B.V., Delft, The Netherlands) from the eddy flux tower. We verified CNR4 calibration stability by comparing against other sensors of the same type at sites located not far from the forest site. Channel 1 of the upward-looking multispectral sensor did not give correct observations. Its values were hence replaced with appropriately-scaled values of Channel 2. The validity of this approach was assessed both with hyperspectral observations at the same site and comparable multispectral observations at a different site [23].

Hyperspectral data were normalized by integration time, and the electronic dark current was subtracted before correction. Hyperspectral observations were transformed into multispectral signals by multiplying them with the corresponding spectral response curves of the multispectral sensor and integrating over the wavelengths, for both downwelling irradiance and upwelling radiance before calculating reflectance factors. Measurements are taken three times (hyperspectral sensor) every ten minutes and 60 times within ten minutes (multispectral sensors); we refer to this further as the measurement-cluster. For each cluster, the absolute sum of irradiance change per channel (${\mathrm{Irr}}_{i,\mathrm{change}}$) and the mean irradiance per channel ($\bar{{\mathrm{Irr}}_{i}}$) are calculated. Measurement clusters are rejected if ${\mathrm{Irr}}_{i,\mathrm{change}}$ exceeds 0.02 $\times \;\bar{{\mathrm{Irr}}_{i}}$ (hyperspectral data) or $\bar{{\mathrm{Irr}}_{i}}$ (multispectral data). Hence, measurements are selected that have been acquired under steady conditions, i.e., mainly clear sky conditions. Subsequently, only measurements between 11:00 a.m. and 01:00 p.m. UTC are considered. Non-vegetational effects usually decrease NDVI values, but highly diffuse illumination conditions illuminating shaded areas and therefore producing directional effects, for example, might also increase NDVI values. Such effects are rather difficult to eliminate. Here, we used sunshine pyranometer data (SPN1, Delta-T Devices Ltd., Cambridge, U.K.) to exclude measurements with a direct to diffuse solar radiation ratio (further referred to as DDSRR) lower than the empirically determined value of 3, to exclude the effects of varying illumination conditions that can affect NDVI [32]. Only upwelling radiance measurements made simultaneously to selected downwelling irradiances were used in order to compute reflectance factors: ${\mathrm{Refl}}_{i}$, *i* = 1,…,4 (see Equation (1)), where π stands for corrections of the hemispheric angle [33].

(1) $${\mathrm{Refl}}_{i}=\pi \times \frac{{\mathrm{Rad}}_{i}}{{\mathrm{Irr}}_{i}}$$

Mean reflectance factors of each measurement cluster serve the generation of NDVI data with $\mathrm{RED}={\mathrm{Refl}}_{1}$ and $\mathrm{NIR}={\mathrm{Refl}}_{4}$ according to Equation (2). The resulting datasets are further referred to as ${\mathrm{NDVI}}_{\mathrm{multi}}$ and ${\mathrm{NDVI}}_{\mathrm{hyper}}$ and contain 1525 and 1447 data points for ‘Hohes Holz’, respectively.

(2) $$\mathrm{NDVI}=\frac{\mathrm{NIR}-\mathrm{RED}}{\mathrm{NIR}+\mathrm{RED}}$$

### 2.3. Satellite Data and Respective NDVI Products

We used spectral data from MODIS datasets MYD09GQ [34] and MOD09GQ [35] with a spatial resolution of 250 m, as well as Sentinel-2A Level 1C (L1C) data [36] with spatial resolutions of 10 and 20 m. Sentinel-2A L1C data were atmospherically-, terrain- and cirrus-corrected with Sen2Cor (Version 2.3.0) [37] and ATCOR 2/3 (Atmospheric & Topographic Correction for Small FOV Satellite Images, RESE^®^, Version 9.1) [38] in order to generate Level 2A (L2A) data. Both processing tools generate scene classifications to distinguish between clear pixels and clouds, shadows and saturated pixels. We continued the study with ATCOR 2/3 (and without Sen2Cor) due to the higher number of successfully-processed scenes with clear sky conditions. ATCOR 2/3 could process 47 out of 78 scenes without error messages. The remaining scenes could not be processed due to the solar zenith during acquisition exceeding 70${}^{\circ }$, software problems or less than one percent of clear pixels. ATCOR 2/3 detected 22 scenes as ‘clear’ around the site ‘Hohes Holz’, while eleven of them showed clouds or shadows after manual examination.

**Figure 3:**
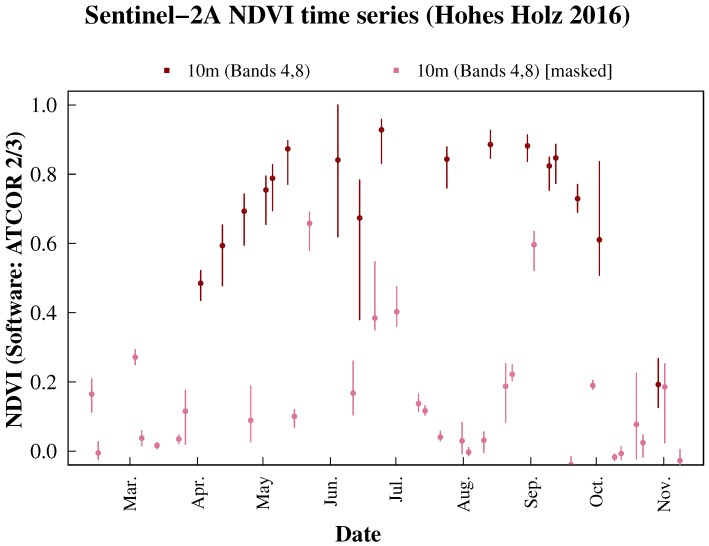
Sentinel-2A NDVI time series from 2016 with 10-m resolution processed with ATCOR 2/3. The lines show the distribution of NDVI values of pixels in an area within 30 m around the eddy flux tower. The dot marks the respective median value. The NDVI of clear pixels are shown in dark colors, whereas pixels with clouds, shadows or saturation are shown in pale colors (masked values).

NDVI (see Equation (2)) is calculated from Bands 1 (RED) and 2 (NIR) of products MYD09GQ and MOD09GQ, resulting in the datasets ${\mathrm{NDVI}}_{\mathrm{aqua}}$ and ${\mathrm{NDVI}}_{\mathrm{terra}}$. Sentinel-2A NDVI is calculated from Bands 4 (RED) and 8 (NIR, 10-m resolution) or 8a (NIR, 20-m resolution) of Sentinel-2A L2A data. These datasets are further referred to as ${\mathrm{NDVI}}_{s2a,10}^{\mathrm{atcor}}$ and ${\mathrm{NDVI}}_{s2a,20}^{\mathrm{atcor}}$, respectively (Figure 3).

### 2.4. NDVI Post-Processing and Phenological Metrics Extraction

Methods for NDVI post-processing and extraction of phenological metrics were implemented in an R-package [39] for phenological data analysis called ‘phenex’ [40]. This includes the Best Index Slope Extraction (BISE) [21] for preprocessing and methods to model phenological time series.

BISE is a dynamic filter algorithm for time series. Starting at the first date of the time series (i.e., DOY = 1 for each year), BISE accepts the next data point if it has a higher value than the previous one. Lower values are accepted if there is no point in a pre-defined time period (‘sliding period’) with a higher value. Higher values are rejected if they exceed a pre-defined threshold ${\mathrm{thres}}_{\mathrm{inc}}$, i.e., the allowed increase of NDVI per day. NDVI fluctuations from natural surfaces greater than 0.1 per day are usually attributed to data errors.

We applied BISE on ${\mathrm{NDVI}}_{\mathrm{aqua}}$ and ${\mathrm{NDVI}}_{\mathrm{terra}}$ with a sliding period of 30 days and an allowed NDVI increase of 10% per day (${\mathrm{thres}}_{\mathrm{inc}}$ = 0.1) in order to remove outliers and eliminate non-vegetational effects, e.g., cloud cover. The resulting datasets were stored as ${\mathrm{NDVI}}_{\mathrm{aqua}}^{\mathrm{bise}}$ and ${\mathrm{NDVI}}_{\mathrm{terra}}^{\mathrm{bise}}$.

The next step is the reconstruction of daily NDVI values based on BISE-selected NDVI observations (${\mathrm{NDVI}}_{\mathrm{multi}}$, ${\mathrm{NDVI}}_{\mathrm{hyper}}$, ${\mathrm{NDVI}}_{\mathrm{aqua}}^{\mathrm{bise}}$, ${\mathrm{NDVI}}_{\mathrm{terra}}^{\mathrm{bise}}$ and ${\mathrm{NDVI}}_{s2a,10}^{\mathrm{atcor}}$). Here, we used linear interpolation; however, other approaches such as fitting a Gaussian function are also commonly used [8]. Phenological metrics extraction is subsequently conducted on the reconstructed NDVI profile and includes green-up and senescence dates, as well as maximum and minimum NDVI and their date of occurrences. Green-up and senescence dates are defined as dates when NDVI values reach a threshold of 55% between the minimum and maximum NDVI values [40,41]. The threshold was determined empirically by comparing satellite-derived green-up dates from deciduous broadleaf forests all over Germany to respective ground observations of the German Weather Service (Deutscher Wetterdienst, DWD) acquired from the Plant-Phenological Online Database (PPODB, www.phenology.de [42]). This database provides around 1500 observations per species, phenological phase and year. The standard deviation of phenological metrics was computed by varying the threshold (normally distributed) according to the satellite product error along the modeled NDVI curve.

## 3. Results

### 3.1. The NDVI Products at Different Scales

**Figure 4:**
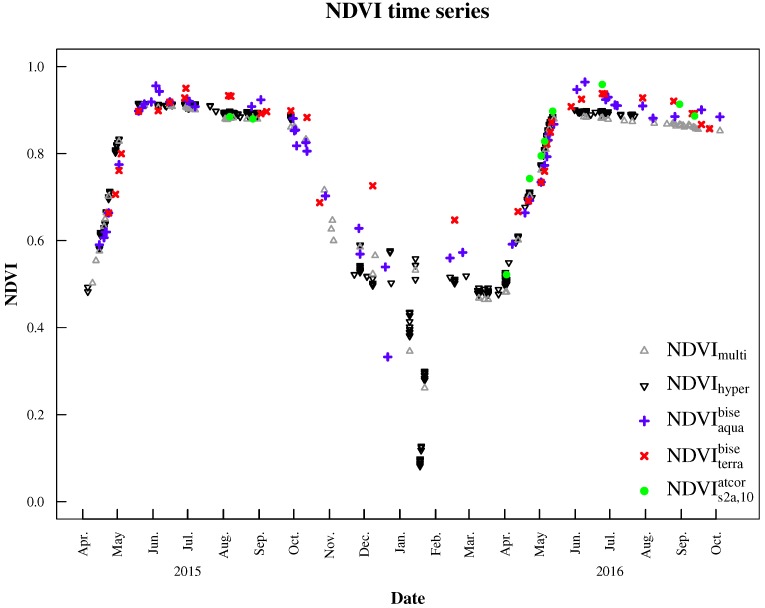
NDVI time series derived from the hyperspectral sensor system (${\mathrm{NDVI}}_{\mathrm{hyper}}$, black triangles), multispectral sensor system (${\mathrm{NDVI}}_{\mathrm{multi}}$, grey triangles), MODIS Aqua (${\mathrm{NDVI}}_{\mathrm{aqua}}^{\mathrm{bise}}$, blue plus signs) and Terra (${\mathrm{NDVI}}_{\mathrm{terra}}^{\mathrm{bise}}$, red crosses), as well as Sentinel-2A (10 m, Bands 4 and 8) processed with ATCOR 2/3 (${\mathrm{NDVI}}_{s2a,10}^{\mathrm{atcor}}$, green filled circles).

NDVI products across all systems showed very similar patterns at the forest site (Figure 4). The temporal NDVI evolution describes precisely the intra-annual patterns of a (changing) canopy structure in deciduous broadleaf forests in temperate climates. Species phenology is mainly driven by temperature (and by day length to a lesser degree) [7] and can be discriminated into two phases: a phase of active/green vegetation (leafy season) from spring to fall and a phase of vegetation in-activity (dormancy) during late autumn and winter. Two distinct phenological events precede these phases: the bud burst event, tightly coupled with leaf development and maturation, as well as the onset of leaf coloring and leaf fall in autumn.

At the start of the season around April, NDVI is increasing until it reaches a maximum in June. The NDVI slightly decreases during the vegetation period and rapidly decreases in fall around mid-September due to senescence. It reaches its minimum value in December. The signal-to-noise-ratio decreases in winter due to snow cover, low irradiance and minor vegetation cover. Sentinel-2A images were manually examined in order to identify misclassified values and excluded if corresponding shortwave pyranometer measurements showed DDSRR < 4. Furthermore, tests demonstrated the applicability of BISE for the removal of these data points. NDVI observations falsely labeled as ‘clear’ by the processing software (and hence exhibiting lower values compared to a true clear pixel within a vegetation period) could be removed by the dynamic filtering procedure.

**Figure 5:**
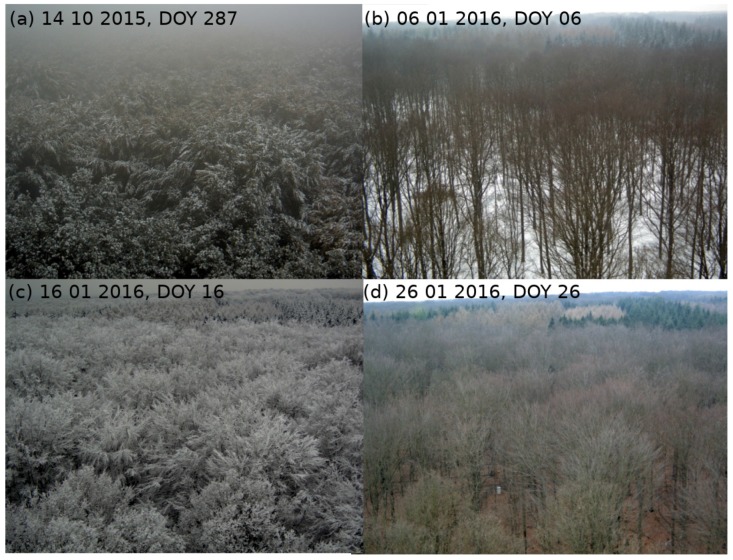
Pictures of the area around the eddy flux tower ‘Hohes Holz’. (**a**–**d**) are captured at different dates, ranging from 14 October 2015–26 January 2016. (**a**) shows the canopy covered by snow and ice in fall 2015. (**b**) shows snow cover of soil seen through a leafless tree canopy. In (**c**), the tree canopy is covered by ice, while (**d**) shows the area after a melting event (no snow or ice left).

Green-up in 2015 and the peak of the vegetation period could be clearly identified by both MODIS NDVI and ground-based datasets. ${\mathrm{NDVI}}_{\mathrm{multi}}$, ${\mathrm{NDVI}}_{\mathrm{hyper}}$, and MODIS NDVI datasets showed the anticipated decrease during summer, where leaf color usually changes from light to dark green. Sentinel-2A data of 2015 were in line with ${\mathrm{NDVI}}_{\mathrm{multi}}$. Fall of 2015 was characterized by unstable illumination conditions (clouds, rain events, snow; see Figure 5a), which led to large gaps in satellite and hyperspectral data. In winter 2015/2016, ${\mathrm{NDVI}}_{\mathrm{terra}}^{\mathrm{bise}}$ showed slightly higher values than ${\mathrm{NDVI}}_{\mathrm{multi}}$ and ${\mathrm{NDVI}}_{\mathrm{hyper}}$. NDVI curves exhibited a strong NDVI decrease in December 2015 and January 2016. This period was characterized by temperatures below 0 ${}^{\circ }$C and precipitation events, with snow/ice cover as the main factor of decreased NDVI values (Figure 5b–d, confirmed by respective temperature and precipitation measurements at the tower site, which are not shown here). Data from this period were excluded from further analysis.

The initial NDVI increase in 2016 (March) was captured by all systems almost simultaneously. ${\mathrm{NDVI}}_{\mathrm{multi}}$, ${\mathrm{NDVI}}_{\mathrm{hyper}}$ and ${\mathrm{NDVI}}_{s2a,10}^{\mathrm{atcor}}$ started with lower NDVI values than ${\mathrm{NDVI}}_{\mathrm{aqua}}^{\mathrm{bise}}$ and ${\mathrm{NDVI}}_{\mathrm{terra}}^{\mathrm{bise}}$. NDVI evolution over the course of leaf unfolding/leaf expansion between April and May was tracked similarly by all employed sensors. Peak NDVI was reached in June, with ${\mathrm{NDVI}}_{\mathrm{aqua}}^{\mathrm{bise}}$ showing the highest and ${\mathrm{NDVI}}_{\mathrm{multi}}$ showing the lowest values. The anticipated slight decrease over the summer months was detected by all sensor systems.

**Figure 6:**
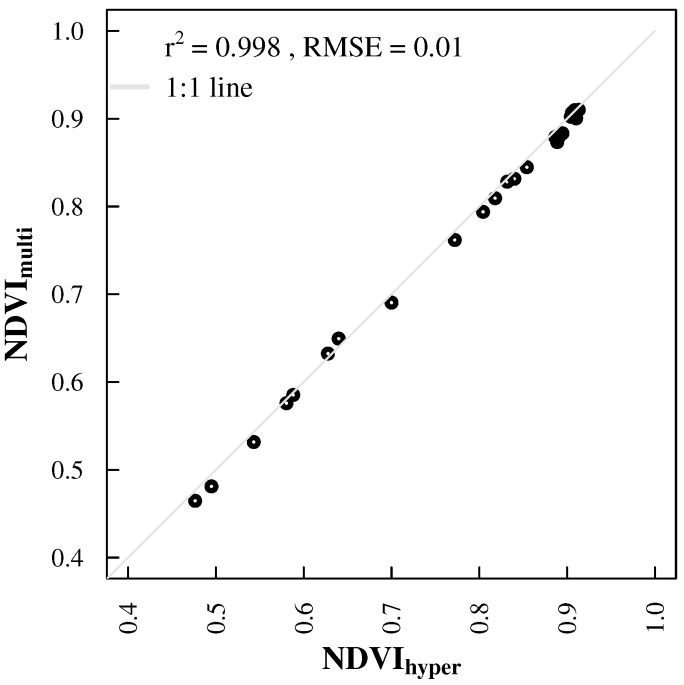
Scatter-plot of NDVI derived from the hyperspectral sensor system (${\mathrm{NDVI}}_{\mathrm{hyper}}$) vs. NDVI from the multispectral sensor system (${\mathrm{NDVI}}_{\mathrm{multi}}$).

**Figure 7:**
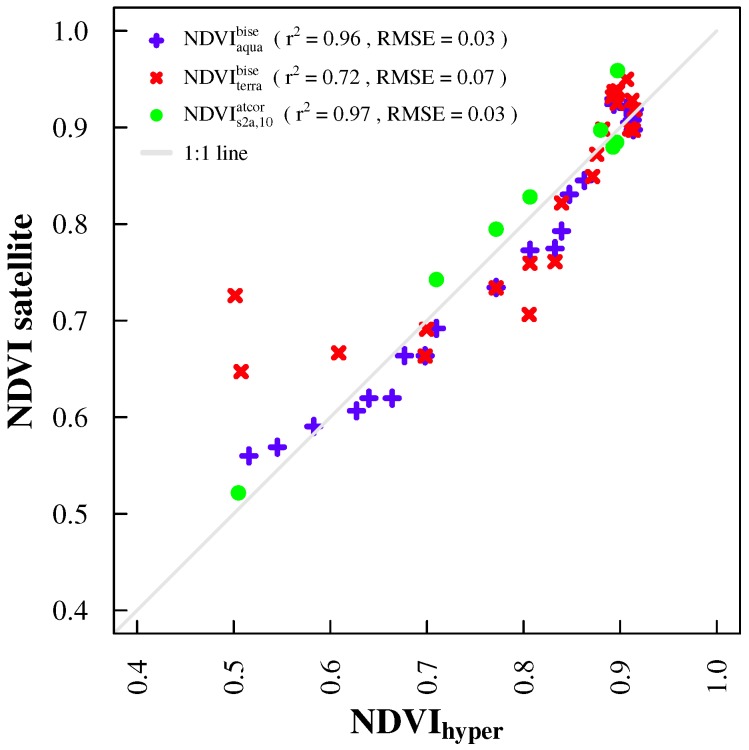
Scatter-plot of NDVI derived from the hyperspectral sensor system (${\mathrm{NDVI}}_{\mathrm{hyper}}$) and satellite NDVI products (NDVI satellite). Satellite NDVI products include ${\mathrm{NDVI}}_{\mathrm{aqua}}^{\mathrm{bise}}$ (blue), ${\mathrm{NDVI}}_{\mathrm{terra}}^{\mathrm{bise}}$ (red), as well as ${\mathrm{NDVI}}_{s2a,10}^{\mathrm{atcor}}$ (green). ${\mathrm{NDVI}}_{\mathrm{multi}}$ and ${\mathrm{NDVI}}_{s2a,20}^{\mathrm{atcor}}$ are not shown due to large similarities with ${\mathrm{NDVI}}_{\mathrm{hyper}}$ and ${\mathrm{NDVI}}_{s2a,10}^{\mathrm{atcor}}$, respectively.

Temporal mismatches between sensor systems reduced the amount of available data points for inter-sensor comparisons (Figure 4 versus Figure 6 and Figure 7). NDVI products from ground spectral measurements correlated well (r${}^{2}$ = 0.998 and RMSE = 0.01; Figure 6) and showed very similar patterns (Figure 4). Correlations of both ground sensors with satellite NDVI products are similar (Table 2) and strong (0.72 ≤ r${}^{2}$ ≤ 0.97), especially with MODIS Aqua and Sentinel-2A NDVI (Figure 7). The magnitude of ${\mathrm{NDVI}}_{\mathrm{multi}}$ and ${\mathrm{NDVI}}_{\mathrm{hyper}}$ was comparable with satellite NDVI data points. There were some minor deviations during the vegetation period and larger deviations in fall 2015 and winter 2015/2016 (Figure 4). ${\mathrm{NDVI}}_{\mathrm{multi}}$ and ${\mathrm{NDVI}}_{\mathrm{hyper}}$ mainly agreed with ${\mathrm{NDVI}}_{\mathrm{aqua}}^{\mathrm{bise}}$, showing a steady NDVI decrease towards an NDVI of around 0.56, while ${\mathrm{NDVI}}_{\mathrm{terra}}^{\mathrm{bise}}$ showed values greater than 0.7 after November 2015. They are also apparent when looking at the correlations between ground and satellite observations (Figure 7).

### 3.2. Analysis of Phenological Metrics

**Figure 8:**
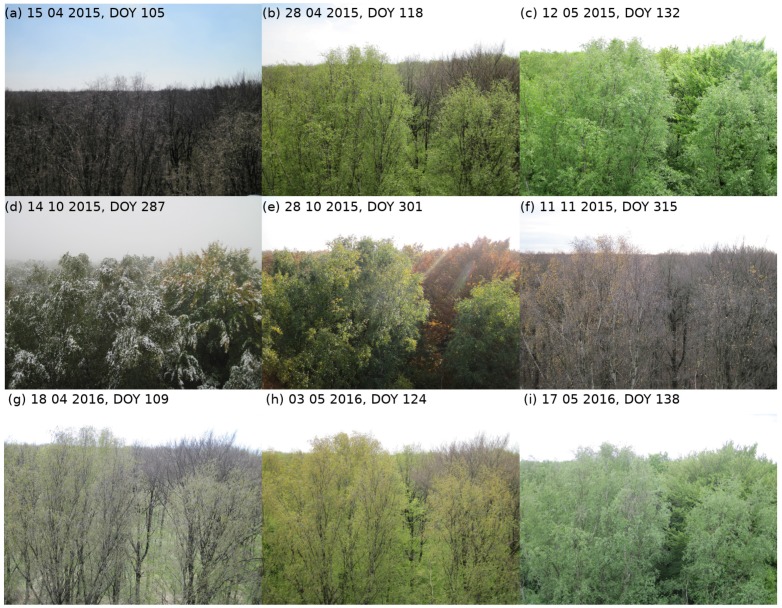
Pictures of phenological state of vegetation around the eddy flux tower ‘Hohes Holz’. (**a**–**i**) are captured at different dates, ranging from 15 April 2015–17 June 2016. (**a**–**c**) show the green-up period in 2015, (**d**–**f**) the senescence period of 2015 and (**g**–**i**) the green-up period of 2016.

Phenological metrics calculated from NDVI time series differed between the sensor systems (Table 3), although the phenological patterns were similar. Green-up dates (GU) in 2015 varied between 25 April (DOY = 115) and 9 May (DOY = 129). GU calculated from ${\mathrm{NDVI}}_{\mathrm{hyper}}$ and ${\mathrm{NDVI}}_{\mathrm{multi}}$ were similar with standard deviations lower than one week. GU calculated from MODIS data were 8–14 days later than GU of in situ and Sentinel-2A data. Calculated green-up dates agreed with observed green-up around the end of April at ‘Hohes Holz’ (Figure 8a–c). Minimum NDVI in 2015 varied between 0.52 and 0.66, depending on the sensor system, and occurred between 5 April (DOY = 95) and 23 April (DOY = 113). Maximum NDVI varied between 0.92 and 0.96 and occurred between 24 May (DOY = 144) and 29 June (DOY = 180). Senescence (SEN) occurred between 13 October (DOY = 286) and 24 October (DOY = 297) and showed a standard deviation of 4.3–12.0 days. SEN calculated from MODIS data occurred around 4–11 days earlier than SEN calculated from in situ and Sentinel-2A data. Calculated senescence dates mainly agreed with observed senescence around the end of October at ‘Hohes Holz’ (Figure 8d–f).

GU varied between 23 April (DOY = 114) and 8 May (DOY = 129) in 2016 with standard deviations lower than one week. GU calculated from ${\mathrm{NDVI}}_{\mathrm{hyper}}$ and ${\mathrm{NDVI}}_{\mathrm{multi}}$ differed by approximately one day. GU from ${\mathrm{NDVI}}_{s2a,10}^{\mathrm{atcor}}$ occurred 5–6 days later, whereas GU from MODIS data occurred 13–16 days later. Calculated green-up dates agreed with observed phenology at ‘Hohes Holz’ (Figure 8g–i). Minimum NDVI varied between 0.49 and 0.65 and occurred between 13 February (DOY = 44) and 2 April (DOY = 93). The latter minimum NDVI was the first observation with clear conditions of Sentinel-2A in 2016. Maximum NDVI varied between 0.89 and 0.96 and occurred between 31 May (DOY = 152) and 24 June (DOY = 176). Calculated phenological metrics mainly agreed with each other and showed standard deviations consistent with the natural intra- and inter-annual phenological variability in the order of several weeks [12,43].

## 4. Discussion

We validated different satellite phenology products by comparing them to multi-sensor ground measurements. The approach relates to Land Product Validation Stage 1 of CEOS (Committee on Earth Observation Satellites) [2]. This required the establishment of a validation site equipped with unattended multispectral and hyperspectral sensor systems for continuous vegetation monitoring. The DFOV systems enable reflectance factor acquisition with (near-)simultaneous measurements of downwelling irradiance and upwelling radiances at high frequency. The approach chosen in this study with a single-sensor DFOV hyperspectral system reduces the costs and effort compared to a system with two spectrometers since an additional permanently-installed spectrometer is not needed [6]. Considerable instability in the scaling of the hyperspectral reflectance factor was observed when comparing time series of hyperspectral with multispectral observations. However, the scaling factor was observed to be independent of wavelength and thus does not affect NDVI, as wavelength independent scaling factors cancel out. For other applications requiring correct scaling of the hyperspectral reflectance factor, a further correction of the calibration instabilities is required. An approach to achieve this will be described in a separate publication. It has also been shown that multispectral data accuracy can potentially be improved by conducting more frequent in situ calibration/validation measurements [44].

The high correlation of NDVI time series between sensors suggests that the multispectral sensor system is sufficient for phenological pattern analysis. The additional use of a hyperspectral system provides, however, information beneficial for the validation of other remote sensing products [6] such as chlorophyll content and leaf area index [45], as well as the possibility to model, for example, plant productivity [3,46]. Multispectral information (of different central wavelengths and FWHM) can further be generated from hyperspectral signals so that the latter consequently enables the validation of different optical satellite missions. Upcoming hyperspectral satellite missions, e.g., EnMap [47], and airborne hyperspectral campaigns will benefit largely from validation sites equipped with spectrometer systems.

Differences in NDVI products are either introduced from sensor specifications (e.g., band configuration), angular effects, measurement scales (and therefore, different observed vegetation patches), calibration accuracy of satellite sensors and atmospheric correction, including cloud-detection [48]. While sensor specifications are mainly comparable, scales and observed vegetation patches differed significantly. Due to the MODIS coarse resolution of 250 m, analysis is limited to only one pixel, whereas we could statistically analyze a cluster (within 30 m around the eddy flux tower) of Sentinel-2A pixels with 10-m resolution for this study. Hence, the observed area had a size of around 62,500 m${}^{2}$ (MODIS) and around 3600 m${}^{2}$ (Sentinel-2A), respectively, compared to the observed ground area of around 72 m${}^{2}$. We observed varying performance of Sentinel-2 processors especially in the presence of small cloud patches, cloud shadows and haze. These problems may be enhanced for MODIS pixels due to their coarser resolution and subsequently more difficulties in the detection of small-scale cloud cover. The angular configuration is different for the sensor systems: MODIS Terra and Aqua overflights differ in solar zenith angle (around five to eight degrees) due to their overpass times around 10:30 a.m. and 01:30 p.m., respectively. ${\mathrm{NDVI}}_{\mathrm{hyper}}$ and ${\mathrm{NDVI}}_{\mathrm{multi}}$ exhibit different illumination angles, introduced by the selection of the time period 11:00 a.m.–01:00 p.m. and DDSRR-filtering. Sentinel-2 observed our region of interest around 10:30 a.m. with a solar zenith angle similar to MODIS Terra. Hence, angular differences can be introduced by both temporal mismatches and angular configuration, which are difficult to differentiate. We mainly observed small NDVI differences within one day between sensor systems, although MODIS Terra showed larger differences to the other sensors during winter 2015/2016. We found considerably higher NDVI values in diurnal multispectral NDVI profiles before 10:30 a.m. only during winter time, assuming potential effects of illumination geometries on the signal. Consequently, MODIS Terra and potentially Sentinel-2 are more sensitive to this effect than MODIS Aqua due to their overpass time before noon. We could not substantiate this effect for Sentinel-2 data, since no observations with clear sky conditions were available for this period. Temporal mismatches between different systems increase using filter algorithms due to the selection of the ‘best’ or ‘true’ NDVI values. Remaining NDVI values after dynamic filtering are used as input for models restoring the phenological profile. Hence, the detection of phenological local extrema, e.g., date and magnitude of the peak of the growing season, is critical to robustly depict phenological metrics. Despite the above described effects, we could not detect a considerable influence of temporal mismatches within one day on phenological metrics extraction. This might be different for other vegetation types or climates with modified phenological timing. Temporal mismatches of several days can in extreme cases cause a derivation of potentially wrong phenological phases, e.g., Sentinel-2A observing clouds during green-up, while MODIS with higher repetition rate observes clear sky conditions in between.

In situ and satellite-based NDVI time series captured similar phenological patterns despite the numerous influencing factors. Correlations between the sensor systems were strong, although some deviations occurred during fall and winter between NDVI of ground sensor systems and MODIS Aqua and Terra. RMSEs between satellite and in situ data exhibited the same magnitude as found in another recent study [49] and as the MODIS NDVI uncertainty (0.02+0.02×NDVI [48]).

Depicted phenological metrics from both in situ datasets were consistent with each other and agreed with Sentinel-2A-derived metrics in 2016. Standard deviations and even the differences of green-up and senescence between the sensor systems were consistent with the natural variance of phenology. MODIS NDVI values during spring and fall were lower compared to in situ data, leading to a deviation in depicted phenological phases: green-up occurred later and senescence earlier. The later GU is contrary to the results found in [4]. Tests with NDVI from hyperspectral data resampled to MODIS bands demonstrated a small shift towards later GU. A larger shift was introduced when averaging NDVI of all Sentinel-2 pixels within the MODIS pixel, although the area is characterized by a homogeneous deciduous broadleaf forest with small clearings. Since satellite observations integrate phenological signals from different species within the pixel, the observed effect of spatial scale mainly stems from different species composition. The species’ signals might also be differently weighted according to the sensors’ point spread function. This hypothesis is supported by high satellite NDVI values in summer, later green-up and day of maximum NDVI, as well as earlier senescence with increasing scale.

Sentinel-2A images are currently available as Level 1C products. Atmospheric, cirrus and terrain corrections are necessary for further analysis. The processing tool ATCOR 2/3 produced reasonable Level 2A products. Although ATCOR 2/3 provided scene classification information supporting the elimination of outliers, misclassifications compromised the resulting NDVI time series. Three approaches can be distinguished eliminating faulty records in time series: images can be manually examined, but this is not feasible for long time series or large datasets. The usage of diffuse shortwave pyranometer measurements as selection criteria is also limited to observation sites with respective infrastructure, but these measurements are getting more and more common. Dynamic filter algorithms, such as BISE, reduce the risk of including false observations while being applicable to large datasets without the need for auxiliary site-specific measurements. Here, BISE was able to consistently remove faulty NDVI values. Daily MODIS NDVI values of 250 m lack scene classification information and respective analysis consequently requires the use of filter algorithms. BISE was able to restore the NDVI profile, although we detected mismatches with ground data during fall 2015 and winter 2015/2016. During December 2015 and January 2016, low temperatures, precipitation events and subsequently snow cover and frozen surfaces led to a strong decrease in multispectral and hyperspectral, as well as MODIS Aqua NDVI not related to vegetation. A strong increase in NDVI during the snow-melt period was detected, which is consistent with other studies observing NDVI of snow-covered vegetation [11,19]. The length of this situation exceeded the sliding period of BISE parametrization. Consequently, decreased MODIS Aqua NDVI values were not eliminated. In contrast, MODIS Terra NDVI increased during winter 2015/2016, relating to the previously-mentioned angular effects.

Apart from assessing the accuracy and precision of satellite products, a validation site offers the opportunity to examine data gaps or periods with low signal-to-noise-ratio introduced by bad illumination conditions and cloud or snow cover. Hence, complete ground NDVI time series are beneficial. Future studies may use a combination of DDSRR-filtering and dynamic filtering, subsequently lowering the DDSRR-threshold to obtain more data points under overcast or hazy conditions, increasing the number of usable ground measurements. The comparison of filtered satellite-based time series with in situ data provides the possibility to modify BISE parameters for satellite phenology depiction, if necessary [50].

## 5. Conclusions

Here, we validated satellite phenology products for a deciduous broadleaf forest. Phenological metrics were consistent between sensor systems and agreed with the natural phenological variance also with sparse data coverage during autumn and winter time. Methods taking the effects of strongly diffuse illumination into account were already addressed in other studies [51,52]. In our measurements, however, we mostly excluded periods of time with highly diffuse illumination because of unstable measurements. A larger number of measurements under highly diffuse and low illumination conditions could be retained by continuous integration time optimization (low illumination) [27,52] or averaging over a larger number of consequent measurements (diffuse conditions). Further, spectral sensors with technical specifications such as research-grade instruments of the WMO (World Meteorological Organization) class quality are beneficial for dealing with these suboptimal conditions. Validation sites should utilize either multispectral sensors with bands chosen according to satellite sensors’ channels or spectrometers simulating respective bands, since the band configuration has an effect on extracted phenological metrics. Improvements in observing the ‘true’ phenological signal in phenological time series were detected by the use of Sentinel-2A data compared to MODIS. Nevertheless, despite the long service and comparably coarse spatial resolution of MODIS Aqua and Terra, MODIS-based phenological metrics agreed fairly well with ground based products. The high spatial resolution of Sentinel-2 reduces mixed-pixel effects ubiquitous in phenology research and offers new opportunities to understand connections between site measurements and large-scale processes, e.g., in the analysis of eddy covariance footprints or vegetation-dependent processes in heterogeneous landscapes. Temporal mismatches between phenological phases and timing of Sentinel-2 observations, under certain conditions critical for the extraction of phenological metrics, will decrease with the upcoming availability of Sentinel-2B data and hence increased temporal resolution of Sentinel-2. The Sentinel-2 processing tool ATCOR 2/3 allows for atmospheric, terrain and cirrus correction of Sentinel-2 images and provides scene classification information on cloud cover, illumination condition and saturation. Despite these comprehensive ancillary data, dynamic filtering algorithms like BISE are still beneficial to generate reliable phenological time series.

## Figures and Tables

**Table 1:** Configuration of the multispectral sensor. Central wavelengths (CW) and the full-width at half-maximum (FWHM) bandwidth are given for each band.

Band	CW (nm)	FWHM (nm)
1	654	37.50
2	708	8.75
3	739	9.50
4	858	9.50

**Table 2:** Squared Pearson correlation coefficients of NDVI product combinations from different sensors for the forest site ‘Hohes Holz’.

	Multispectral	MODIS Aqua	MODIS Terra	Sentinel-2A
**Hyperspectral**	0.998	0.96	0.72	0.97
**Multispectral**	1	0.97	0.72	0.97
**MODIS Aqua**		1	0.77	-

**Table 3:** Phenological metrics extracted from the NDVI time series with the R-package ‘phenex’. These metrics include the Day Of the Year (DOY) of green-up, senescence and respective standard deviations (sd), minimum and maximum NDVI, as well as the DOY of their occurrences. A dash (-) indicates if the metric could not be extracted from the specific time series due to unavailable data.

	Hyperspectral	Multispectral	MODIS Aqua	MODIS Terra	Sentinel-2A
2015/2016					
**DOY Green-up**	115/115	115/114	123/127	129/129	-/118
**sd(Green-up)**	2.1/5.2	2.4/4.3	3.8/3.2	6.4/3.3	-/5.2
**DOY min(NDVI)**	95/77	99/74	105/44	113/48	-/93
**DOY max(NDVI)**	166/152	144/160	154/161	180/176	-/176
**min(NDVI)**	0.49/0.49	0.50/0.46	0.54/0.56	0.66/0.65	-/0.52
**max(NDVI)**	0.92/0.90	0.91/0.88	0.96/0.96	0.95/0.94	-/0.96
**DOY Senescence**	295/-	297/-	291/-	286/-	-/-
**sd(Senescence)**	5.4/-	4.2/-	6.8/-	12.4/-	-/-
